# Association of serum angiopoietin-2 with malnutrition, inflammation, atherosclerosis and valvular calcification syndrome and outcome in peritoneal dialysis patients: a prospective cohort study

**DOI:** 10.1186/s12967-018-1687-0

**Published:** 2018-11-16

**Authors:** Xiaoxiao Yang, He Zhang, Yuanyuan Shi, Zanzhe Yu, Hao Yan, Zhaohui Ni, Jiaqi Qian, Wei Fang

**Affiliations:** 1 Department of Nephrology, Renji Hospital, School of Medicine, Shanghai Jiao Tong University, No. 160, Pujian Road, Pudong District, Shanghai, 200127 People’s Republic of China; 2 Shanghai Center for Peritoneal Dialysis Research, Shanghai, People’s Republic of China

**Keywords:** Angiopoietin-2, Atherosclerosis, Calcification, Inflammation, Malnutrition, Peritoneal dialysis

## Abstract

**Background:**

To examine serum angiopoietin-2 (Angpt-2) in relation to malnutrition, inflammation, atherosclerosis and cardiac valvular calcification, so-called MIAC syndrome and its predictive role in outcomes of peritoneal dialysis (PD) patients.

**Methods:**

A prospective observational study was conducted in 324 chronic PD patients. Biochemical analysis was performed at baseline for serum angiopoietins, albumin and high sensitive C-reactive protein (hs-CRP) and echocardiography was done to detect cardiac valvular calcification. Primary study end points were fatal or nonfatal cardiovascular events and mortality.

**Results:**

The median of serum Angpt-2 levels was 5.44 ng/mL (interquartile range, 3.41–7.85). Across the three tertiles of serum Angpt-2, a significant trend effect was observed for body mass index, normalized protein catabolic rate, calcium × phosphorus product, hs-CRP, brain natriuretic peptide, lower-density lipoprotein cholesterol, left ventricular ejection fraction, total weekly urea clearance and residual renal function (all *p* < 0.05). Serum Angpt-2 showed a significant increase across the four groups of patients with increasing components of MIAC syndrome (*p* < 0.001). There were 77 deaths and 57 cardiovascular events. High serum Angpt-2 was an independent predictor of fatal and nonfatal cardiovascular events in PD patients (*p* = 0.02), however serum Angpt-2 was not an independent predictor of all-cause mortality (*p *= 0.3).

**Conclusions:**

Serum Angpt-2 showed close association with valvular calcification, atherosclerosis, inflammation and malnutrition, having significant independent prognostic value and is useful for cardiovascular event stratification in chronic PD patients. Angpt-2 might be a potential mediator of increased cardiovascular risk in patients undergoing PD treatment.

**Electronic supplementary material:**

The online version of this article (10.1186/s12967-018-1687-0) contains supplementary material, which is available to authorized users.

## Background

Overall mortality rates among end-stage renal disease (ESRD) patients have consistently declined over the last 15 years, but dialysis patients continue to have substantially higher mortality compared to the general population and nearly half of the deaths are accounted for by cardiovascular causes [1]. It is widely recognized that dialysis patients are at higher risk of developing accelerated cardiovascular diseases (CVD), resulted from a clustering of both traditional and non-traditional risk factors. It has been reported that an elevation of either C-reactive protein (CRP) or interleukin-6 (IL-6), as a biomarker of systemic inflammation, was strongly associated with CVD in peritoneal dialysis (PD) patients [2], as well as risk factors of both all-cause and cardiovascular mortality [3–5]. Malnutrition assessed either by subjective global assessment (SGA) [6] or by serum albumin [7] in PD patients is associated with a statistically significant increase in mortality. There is a tight link among malnutrition, inflammation and atherosclerosis (MIA syndrome) in chronic renal failure patients [8] as well as dialysis patients [9]. It had been reported that more than 80% of young patients on dialysis had extensive coronary artery calcification correlated with the duration of dialysis [10]. Cardiac valvular calcification (VC) is a powerful predictor for mortality and cardiovascular deaths in long-term dialysis patients [11]. Previous study demonstrated a strong association of inflammation and malnutrition with cardiac VC, similar to that of atherosclerosis, which was together called as MIAC syndrome [12].

Angiopoietin-1 (Angpt-1) and angiopoietin-2 (Angpt-2) are ligands of the Tie-2 receptor, a family of growth factors specific for the vascular endothelium [13–15]. Angpt-1-mediated Tie-2 activation is required to maintain quiescent endothelium, while Angpt-2 destabilizes quiescent endothelium. In addition to angiogenesis, Fiedler et al. [16, 17] had found that Angpt-2 might play an effective role in regulating endothelial cell inflammatory response, thereby exerting a permissive role for the activities of pro-inflammatory cytokines. Circulating Angpt-2 level was increased in chronic kidney disease (CKD) patients [18] as well as in patients undergoing dialysis treatment and individual Angpt-2 levels significantly decreased within 3 months after kidney transplantation [19]. One explanation for increasing Angpt-2 level in CKD patients with or without dialysis treatment might be the high inflammatory status among these patients, furthermore increased Angpt-2 expression has been observed on stimulation with high glucose and tumor necrosis factor-α levels, both frequently observed in dialysis patients [16, 20]. Angpt-2 might be a mediator (and thus a marker) that accounts for accelerated atherosclerosis in dialysis patients [19, 21]. However, high risk of the cardiovascular event as well as cardiovascular mortality in PD patients is still an unsolved question.

With this background, we examined serum Angpt-2 in relation to cardiac valvular calcification, atherosclerosis, inflammation and malnutrition, so-called MIAC syndrome in chronic PD patients, furthermore the predictive role of increased serum Angpt-2 and the MIAC syndrome in the clinical outcomes of chronic PD patients.

## Materials and methods

### Study design

This was a prospective observational study based on a single center in China. Study participants were recruited between January 2014 and April 2015 and prospectively followed up to the end of the study (31 January 2018). All procedures followed in this study were in accordance with the ethical standards of the responsible committee on human experimentation (institutional and national) and with the Helsinki Declaration of 1975. The study protocol was approved by the Human Research Ethics Committee of Renji Hospital, School of Medicine, Shanghai Jiao Tong University. All participants gave their written informed consent.

### Study participants

Patients were eligible for study entry if they had been maintained stably on PD treatment. Exclusion criteria included patients developing acute coronary syndrome, acute heart failure, peritonitis, exit-site infection, or other infective complications, or with underlying active malignancy, chronic liver disease, systemic lupus erythematosus requiring immunosuppression, systemic vasculitis, chronic rheumatic heart disease and congenital heart disease; presence of systemic inflammatory disease; patients who refused to give consent; or patients with incomplete data. Based on inclusion and exclusion criteria, 324 PD patients were recruited in our study, representing 72% of the total PD population in our center. All patients were dialyzed using conventional lactate-buffered glucose- based PD solutions (Dianeal^®^, Baxter, China).

### Clinical and demographic data collection

The following demographic characteristics were collected at study baseline: age, gender, underlying cause of ESRD, duration on dialysis, height, weight, presence of diabetes mellitus (DM) and atherosclerotic vascular disease (AVD). AVD was defined as the presence of ischaemic heart disease, history of angina, previous myocardial infarction, coronary artery bypass surgery or stenting, cerebrovascular event, transient ischaemic attack or peripheral vascular disease with or without amputation. DM was defined either as a comorbid disease or as the etiology of ESRD. Body mass index was calculated as body weight in kilograms divided by height in meters squared.

### Angiopoietin-2 and biochemical parameters assay

At the time of enrollment, fasting venous blood of each patient was collected for the measurement of serum Angpt-1, Angpt-2, soluble Tie-2 (sTie-2). Serum Angpt-1, Angpt-2 and sTie-2 were determined using enzyme linked immunosorbent assay (ELISA) kit (R&D Systems Inc, Minneapolis, MN, USA). All samples were run simultaneously and in duplicate to avoid intra- and inter-assay variations. The following laboratory parameters were also measured: high sensitive C-reactive protein (hs-CRP), serum albumin, hemoglobin, calcium, phosphorus, intact parathyroid hormone (iPTH), lipid profile (which include total cholesterol, lower-density lipoprotein (LDL) cholesterol, high-density lipoprotein (HDL) cholesterol and triglyceride) and brain natriuretic peptide (BNP). hs-CRP was measured using the Tina-quant CRP (Latex) ultra-sensitive assay (D & P modular analyser, Roche Diagnostics GmbH, Mannheim, Germany). Serum albumin was measured using the bromcresol purple method, while total holesterol and triglyceride, using enzymatic assay on the Hitachi 911 analyzer (Roche Diagnostics GmbH). BNP was measured using a fluorescence immunoassay with the Triage BNP Test (Biosite, San Diego, CA). iPTH was determined by chemiluminescence immunoassay on the Immulite analyser (Diagnostic Products Corp., Los Angeles, CA).

### Indices of dialysis adequacy

Patients were asked to collect 24-h urine and dialysate to measure urea and creatinine concentrations. Adequacy of dialysis was determined by measuring total weekly urea clearance (Kt/V) and creatinine clearance (Ccr) using standard methods [22]. Weekly Ccr was normalized to 1.73 m^2^ of body surface area. Contribution of urea clearance by peritoneal dialysis was estimated separately. Residual renal function was calculated as an average of 24-h urine urea and creatinine clearance [23]. Urea nitrogen and creatinine concentrations were determined using enzymatic assay on the Hitachi 911 analyzer (Roche Diagnostics GmbH). Normalized protein catabolic rate (nPCR) was calculated by the methods described by Randerson, Chapman, and Farrell and normalized to standard body weight (total body water/0.58) [24]. Total body water (V) was determined by Watson and Batt’s formula [25].

### Echocardiography

Two-dimensional echocardiography was performed using a Vingmed GE System Sonographic machine. All echocardiographs were performed according to the recommendations of the American Society of Echocardiography [26] and were analyzed by a single experienced cardiac sonographer. Cardiac VC was defined as bright echoes on one or more cusps of more than 1 mm in either mitral or aortic valves or both. Sensitivity and specificity for echocardiographic detection of calcium in both the mitral and the aortic valves have been reported to be 76% and 89–94%, respectively [27]. Left ventricular (LV) mass index was calculated using the modified American Society of Echocardiography cube formula proposed by Devereux et al. [28] and indexed by body surface area.

### Follow-up

All patients were followed up prospectively from the enrollment of the study until death, cessation of PD, transfer to other centers, or to the end of the study (31 January 2018). The outcomes evaluated were death from all causes and fatal or nonfatal cardiovascular events. For patients who developed multiple cardiovascular events, survival analysis was limited to the first event. Cardiovascular event included acute myocardial ischemic event, sustained atrial or ventricular arrhythmia, stroke, peripheral vascular disease and sudden death defined and diagnosed clinically as unexpected natural death within 1 h from symptom onset and without a prior condition that would appear fatal [29, 30]. Acute myocardial ischemia was diagnosed by the attending physician based on the presence of symptoms and serial electro- cardiographic and cardiac enzyme changes in accordance with World Health Organization criteria.

### Statistical analysis

Continuous data were expressed as mean ± SD or median (interquartile range) depending on the distribution of data. Patients were stratified by tertiles of serum Angpt-2 concentration. Comparisons across the tertiles were performed using one-way analysis of variance (ANOVA), Kruskal–Wallis test or χ^2^ test, when appropriate. Cumulative survival curves were generated using the Kaplan–Meier method and log-rank test. In the view of the skewed distribution, serum Angpt-1, Angpt-2, sTie-2, Angpt-1/Angpt-2 ratio were log_10_-transformed before performing comparison among different groups of MIAC syndrome and entering into the Cox analysis. The Cox proportional hazards model was used to estimate the relative risks of all-cause mortality and fatal and non-fatal cardiovascular events for different variables. Basic demographic characteristics and components of MIAC syndrome as well as factors with *p *< 0.05 on univariate Cox analysis for all-cause and fatal and non-fatal cardiovascular events were further entering into the multivariate Cox regression analysis. We plotted scaled schoenfeld residuals versus time for all variables and computed their correlation against time to confirm that all variables considered in the Cox regression analysis met the assumptions of proportional hazards. A *p*-value of < 0.05 was considered to be statistically significant. Statistical analysis was performed using SPSS software, version 11.0 (SPSS, Inc., Chicago, IL).

## Results

### Characteristics of the cohort

A total of 324 patients were enrolled, which consisted of 166 (51%) males with a mean age of 57.4 ± 14.1 years and median PD duration of 31.5 (12.4–57.1) months. The causes of ESRD were chronic glomerulonephritis in 100 patients (30.8%), diabetic nephropathy in 42 patients (13.0%), hypertensive nephrosclerosis in 12 patients (3.7%), obstructive uropathy in 4 patients (1.2%), polycystic kidney disease in 9 patients (2.8%), tubulointerstitial nephritis in 10 patients (3.1%), others 20 patients (6.2%). Underlying renal diagnosis was unknown in 127 patients (39.2%). The detail baseline characteristics of participants were summarized in Table 1.

**Table 1:** Baseline characteristics of the study population

Variables	Total (n = 324)
Age (year)	57.4 ± 14.1
Male gender (n [%])	166 (51%)
Dialysis duration (month)	31.5 (12.4–57.1)
Body mass index (kg/m^2^)	23.2 ± 3.9
Diabetes mellitus (n [%])	84 (25.9%)
Background AVD (n [%])	99 (30.6%)
Background VC (n [%])	67 (22.3%)
Serum albumin (g/L)	37.0 ± 4.6
Hemoglobin (g/L)	107.4 ± 17.0
hs-CRP (mg/L)	2.5 (0.9–6.6)
Ca × P (mg^2^/dL^2^)	58.0 ± 18.8
iPTH (pg/L)	282.0 (134.0–591.0)
Total weekly urine clearance	1.94 ± 0.37
Total weekly creatinine clearance (L/week/1.73 m^2^)	62.0 ± 18.26
Residual renal function (mL/min)	0.92 (0–2.88)

Calcium × phosphorus product in mg^2^/dL^2^ to mmol^2^/L^2^, × 0.0806

*AVD* atherosclerotic vascular disease, *VC* valvular calcification, *hs-CRP* high sensitive C-reactive protein, *iPTH* intact parathyroid hormone, *Ca *×* P* calcium × phosphorus product

### Parameters between different tertiles of circulating angiopoietin-2 level

The median serum Angpt-1, Angpt-2, sTie-2 level of the cohort was 42.0 (28.30–64.82) ng/mL, 5.44 (3.41–7.85) ng/mL and 20.10 (15.26–25.42) ng/mL, respectively. Patients were stratified into three tertiles according to the serum Angpt-2 concentration, namely those with serum Angpt-2 ≤ 3.94 ng/mL (lower tertile), those with serum Angpt-2 between 3.94 and 7.03 ng/mL (middle tertile) and those with serum Angpt-2 ≥ 7.03 ng/mL (upper tertile). The clinical and demographic characteristics of patients across the three tertiles of serum Angpt-2 were detailed in Table 2. A trend effect was observed across the three tertiles of increasing serum Angpt-2 concentration for body mass index, nPCR, calcium × phosphorus product, hs-CRP, BNP, LDL cholesterol, LV ejection fraction, total weekly urine clearance, total weekly creatinine clearance and residual renal function (all *p *< 0.05). No significant difference was observed in the serum albumin across the three tertiles of increasing serum Angpt-2 concentration (all *p* > 0.05).

**Table 2:** Clinical characteristics of patients across tertiles of serum Angpt-2

Variables	Serum Angpt-2 in tertiles			*p* for trend
	Lower (n = 108)	Middle (n = 108)	Upper (n = 108)	
Age (year)	55.2 ± 14.5	57.8 ± 13.5	59.3 ± 14.1	0.1
Male gender (n [%])	56 (52%)	56 (52%)	54 (50%)	0.9
Dialysis duration (mo)	25.6 (3.2–55.1)	34.9 (12.6–60.7)	34.6 (15.1–55.8)	0.3
Diabetes mellitus (n [%])	22 (20.4%)	28 (25.9%)	34 (31.5%)	0.2
Body mass index (kg/m^2^)	22.1 ± 3.8	23.2 ± 3.7	24.3 ± 4.1	< 0.001
Background AVD (n [%])	26 (24.1%)	34 (31.5%)	39 (36.1%)	0.2
Background VC (n [%])	17 (15.7%)	22 (20.4%)	28 (25.9%)	0.2
Hemoglobin (g/L)	107.7 ± 15.7	107.7 ± 16.8	106.7 ± 18.6	0.9
Serum albumin (g/L)	37.0 ± 4.6	37.0 ± 4.5	37.1 ± 4.8	0.9
n PCR	0.93 ± 0.19	0.87 ± 0.19	0.83 ± 0.15	< 0.001
Ca × P (mg^2^/dL^2^)	54 ± 17	58 ± 17	62 ± 22	0.009
iPTH (pg/L)	269 (124.9–568.0)	266.0 (133.3–563.5)	376 (149.8–616.3)	0.3
hs-CRP (mg/L)	1.37 (0.65–3.73)	2.63 (0.89–5.72)	3.7 (1.45–8.38)	< 0.001
BNP (pg/mL)	66.5 (23.0–158.8)	89 (50.5–202.8)	97.5 (44.0–198.8)	0.006
Total cholesterol (mmol/L)	4.77 (4.05–5.40)	5.05 (4.25–5.69)	4.64 (3.97–5.50)	0.08
LDL cholesterol (mmol/L)	2.68 (2.14–3.26)	2.85 (2.24–3.40)	2.56 (1.83–3.07)	0.03
HDL cholesterol (mmol/L)	1.17 (0.92–1.58)	1.16 (0.93–1.50)	1.10 (0.86–1.31)	0.07
Triglyceride (mmol/L)	1.50 (1.13–2.24)	1.67 (1.17–2.46)	1.85 (1.22–3.03)	0.08
LV mass index (g/m^2^)	114.9 (82.2–159.4)	128.3 (91.8–177.5)	136.0 (92.9–189.8)	0.2
LV ejection fraction (%)	67 (64–71)	65.5 (60–69.8)	64 (58–68.5)	0.005
Total weekly urine clearance	2.05 ± 0.41	1.91 ± 0.36	1.86 ± 0.30	0.001
Total weekly creatinine clearance (L/wk/1.73 m^2^)	67.68 ± 21.42	61.47 ± 17.40	56.88 ± 13.61	< 0.001
Residual renal function (mL/min)	1.96 (0.53–4.18)	0.77 (0–2.52)	0.21 (0–1.88)	< 0.001

Calcium × phosphorus product in mg^2^/dL^2^ to mmol^2^/L^2^, × 0.0806

*AVD* atherosclerotic vascular disease, *VC* valvular calcification, *Angpt-2* angiopoietin-2, *nPCR* normalized protein catabolic rate, *hs-CRP* high sensitive C-reactive protein, *Ca* × *P* calcium × phosphorus product, *BNP* brain natriuretic peptide, *LV* left ventricular, *iPTH* intact parathyroid hormone, *LDL* lower-density lipoprotein, *HDL* high-density lipoprotein

### Correlation of circulating angiopoietin-2 level with MIAC syndrome

**Fig. 1:**
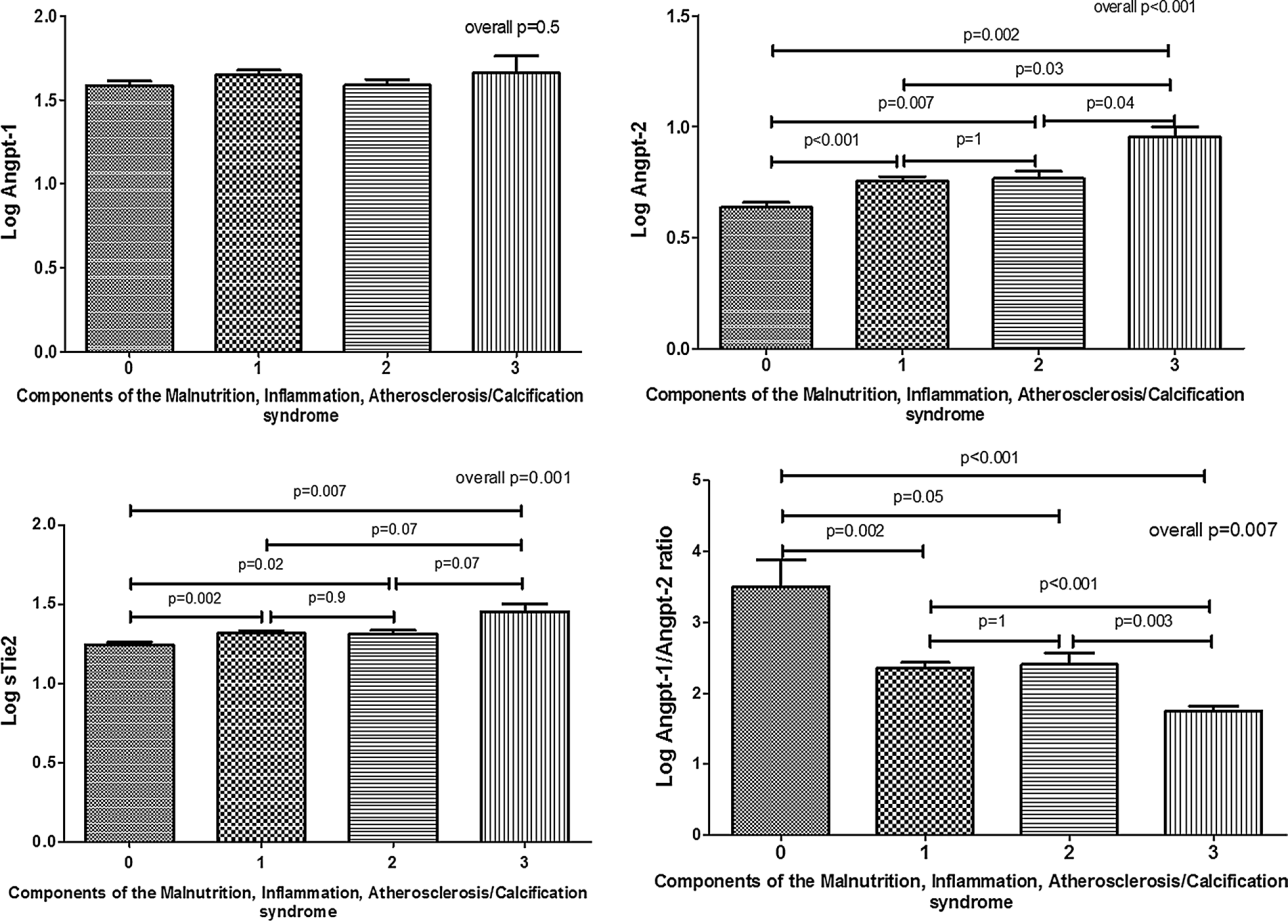
Serum Angpt-1, Angpt-2, sTie-2 concentration and Angpt-1/Angpt-2 ratio in relation to the presence of zero (n = 138), one (n = 118), two (n = 62) and all three (n = 6) components of the malnutrition, inflammation, atherosclerosis/calcification syndrome. Malnutrition was defined as serum albumin < 30 g/L, inflammation was defined as hs-CRP ≥ 5 mg/L, atherosclerosis/calcification was defined by the presence of background AVD or cardiac VC

Serum Angpt-2 differed significantly among the four groups of patients stratified on the basis of the presence or absence of AVD and valvular calcification (overall; *p *= 0.003). Patients with neither AVD nor valvular calcification had the lowest serum log_10_ Angpt-2 while those with both AVD and valvular calcification had higher serum Angpt-2 (*p* = 0.05). Serum Angpt-2 did not differ significantly between patients with either valvular calcification or AVD (*p* > 0.05, Additional file 1: Figure S1A). Patients were also stratified into four groups on the basis of the presence or absence of inflammation and malnutrition. Inflammation was defined as those with hs-CRP ≥ 5 mg/L, while malnutrition was defined as those with serum albumin < 30 g/L. Serum Angpt-2 was significantly different across the four groups of patients stratified on the basis of inflammation and malnutrition (overall; *p* = 0.004). Serum log_10_ Angpt-2 was the highest among patients with both inflammation and malnutrition and lowest among those without inflammation and malnutrition (*p* = 0.01). No significant difference was noted in serum log_10_ Angpt-2 between patients with either inflammation or malnutrition (*p* > 0.05, Additional file 1: Figure S1B). Finally, patients were stratified into four groups on the basis of the presence of zero, one, two and all three components of the malnutrition, inflammation, atherosclerosis/calcification (MIAC) syndrome. Serum log_10_ Angpt-2 showed a significant increase across the four groups of patients with increasing components of the MIAC syndrome (overall; *p* < 0.001). Furthermore, serum log_10_ sTie-2 and log_10_ Angpt-1/Angpt-2 ratio were significantly different among four groups (overall; *p* = 0.001 and *p* = 0.007, respectively). Serum log_10_ sTie-2 was the highest among patients with all three components of MIAC syndrome, while log_10_ Angpt-1/Angpt-2 ratio was the lowest among these patients. There was no significant difference of serum log_10_ Angpt-1 level among four groups (overall; *p *= 0.5) (Fig. 1).

### Circulating angiopoietin-2 level and patients’ outcomes

After follow-up for 31.7 ± 13.5 months, 77 patients (24%) had died and 28 patients (9%) had undergone kidney transplantation, 31 patients (10%) were switched to permanent hemodialysis therapy, 9 patients (3%) had transferred to other centers and 2 patients (0.6%) had out of follow-up (Both of them were out of touch). Causes of death included 9 acute myocardial ischemic events, 20 strokes, 10 sudden deaths, 3 heart failure episodes, 5 peripheral vascular diseases, 6 peritonitis episodes, 12 other infections, 4 malignancies (1 lung carcinoma, 1 breast carcinoma and 2 stomach carcinoma) and 8 patients with etiology unknown. Baseline median serum Angpt-2 level was significantly higher in patients who died versus those who survived during follow-up [6.58 (4.36–9.28) ng/mL versus 5.06 (3.33–7.51) ng/mL, respectively; *p* = 0.002].

Fifty-seven patients experienced one or more cardiovascular events. The first cardiovascular event included 27 strokes, 13 acute myocardial ischemic events, 7 peripheral vascular disease events and 10 sudden cardiac deaths. Baseline median serum Angpt-2 level was significantly higher in patients who had one or more cardiovascular events versus those with no cardiovascular event during follow-up [6.63 (5.05–6.78) ng/mL versus 5.08 (3.32–7.59) ng/mL, *p* < 0.001].

**Fig. 2:**
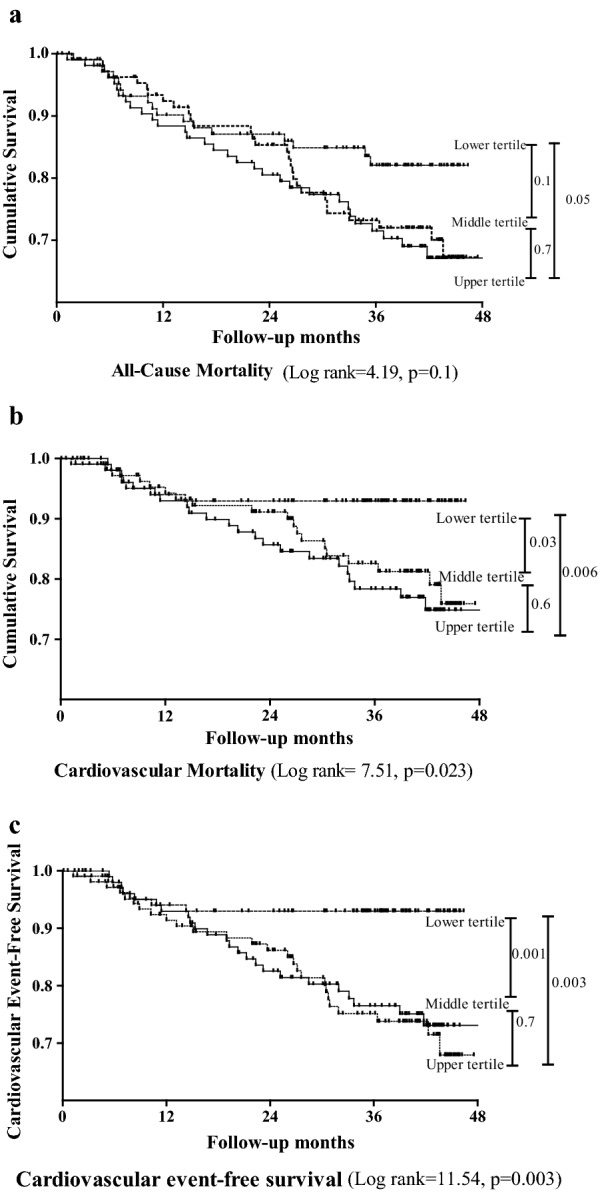
Kaplan-Meier estimates of **a** overall survival probability and **b** cardiovascular mortality **c** fatal or nonfatal cardiovascular event-free survival probability of patients stratified by tertiles of serum angiopoietin-2 (Angpt-2). Lower tertile: Angpt-2 ≤ 3.94 ng/mL; middle tertile: Angpt-2 > 3.94 to < 7.03 ng/mL; upper tertile Angpt-2 ≥ 7.03 ng/mL

According to the Kaplan–meier survival analysis, a significant increase in cardiovascular mortality (log rank = 7.51, *p* = 0.023; Fig. 2b), and fatal or nonfatal cardiovascular event (log rank = 11.54, *p* = 0.003; Fig. 2c) was observed across the three tertiles of increasing serum Angpt-2. While there was no significant difference of all-cause mortality among three tertiles of serum Angpt-2 (log rank = 4.19, *p* = 0.1; Fig. 2a). By univariate Cox analysis, serum Angpt-2 showed a highly significant association with all-cause mortality (*p* = 0.007) as well as fatal and non-fatal cardiovascular events (*p* = 0.001, Table 3).

**Table 3:** Univariate Cox analysis of factors in relation to all-cause mortality and cardiovascular events

	Unite increase	All-cause mortality (n = 77)		Fatal and non-fatal cardiovascular events (n = 57)	
		HR (95%)	*p* value	HR (95%)	*p* value
Age	1 year	1.06 (1.04–1.08)	< 0.001	1.04 (1.02–1.07)	< 0.001
Male gender	–	0.94 (0.60–1.46)	0.8	0.88 (0.53–1.48)	0.6
Diabetes mellitus	–	2.39 (1.52–3.77)	< 0.001	2.29 (1.35–3.89)	0.002
Hypertension	–	4.75 (0.66–34.18)	0.1	3.52 (0.49–25.46)	0.2
Background AVD	–	4.94 (3.10–7.88)	< 0.001	4.59 (2.69–7.85)	< 0.001
Background VC	–	2.02 (1.25–3.26)	0.004	1.33 (0.73–2.41)	0.3
Ca × P	1 mg^2^/dL^2^	1.0 (0.98–1.01)	0.4	1.0 (0.98–1.01)	0.6
iPTH	1 pmol/L	1.0 (0.99–1.0)	0.4	0.99 (0.99–1)	0.2
hs-CRP	1 mg/L	1.02 (1.0–1.03)	0.03	1.01 (0.99–1.03)	0.2
Serum albumin	1 g/L	0.91 (0.86–0.95)	< 0.001	0.91 (0.89–0.97)	0.001
Hemoglobin	1 g/L	1.0 (0.98–1.01)	0.7	1.00 (0.99–1.02)	0.8
LDL cholesterol	1 mmol/L	1.19 (0.99–1.44)	0.06	1.31 (1.09–1.57)	0.004
LV mass index	1 g/m^2^	1.002 (0.99–1.01)	0.2	1.004 (1.0–1.008)	0.05
Log_10_ Angpt-2	1 ng/mL	3.67 (1.43–9.45)	0.007	6.71 (2.15–20.88)	0.001
Residual renal function	1 mL/min	0.93 (0.83–1.04)	0.2	0.97 (0.86–1.10)	0.7

Calcium × phosphorus product in mg^2^/dL^2^ to mmol^2^/L^2^, × 0.0806

*AVD* atherosclerotic vascular disease, *VC* valvular calcification, *Ca* × *P* calcium × phosphorus product, *iPTH* intact parathyroid hormone, *hs-CRP* high sensitive C-reactive protein, *LDL* lower-density lipoprotein, *LV* left ventricular, *Angpt-2* angiopoietin-2

In the multivariate Cox regression analysis, the significance of serum Angpt-2 associated with all-cause mortality in model was lost when adjusting for age, male gender, diabetes and components of MIAC syndrome (hs-CRP, serum albumin, background VC and background AVD) (*p* = 0.3, Table 4). Otherwise, in the multivariate Cox regression models for fatal and non-fatal cardiovascular events, serum Angpt-2 remained an independently predictive factor when adjusting for age, male gender, diabetes, LDL cholesterol, LVMI and components of MIAC syndrome (hs-CRP, serum albumin, background VC and background AVD) (*p *= 0.02, Table 5).

**Table 4:** Multivariate Cox regression models for all-cause mortality [expressed as hazard ratios (95% confidence intervals), *p*-value]

	Unit increase	HR (95%)	*p* value
Age	1 year	1.03 (1.01–1.06)	0.006
Male gender	–	0.86 (0.54–1.37)	0.5
Diabetes mellitus	–	1.28 (0.78–2.10)	0.3
Log_10_ Angpt-2	1 ng/mL	1.69 (0.64–4.47)	0.3
hs-CRP	1 mg/L	1.01 (0.99–1.03)	0.4
Serum albumin	1 g/L	0.93 (0.88–0.99)	0.02
Background VC	–	1.05 (0.61–1.80)	0.9
Background AVD	–	2.93 (1.76–4.88)	< 0.001

*AVD* atherosclerotic vascular disease, *VC* valvular calcification, *hs-CRP* high sensitive C-reactive protein, *Angpt-2* angiopoietin-2

**Table 5:** Multivariate Cox regression models for ftal and non-fatal cardiovascular events [expressed as hazard ratios (95% confidence intervals), *p*-value]

	Unit increase	HR (95%)	*p* value
Age	1 year	1.03 (0.99–1.06)	0.07
Male gender	–	0.52 (0.25–1.08)	0.08
Diabetes mellitus	–	1.11 (0.59–2.09)	0.7
LDL cholesterol	1 mmol/L	1.06 (0.78–1.45)	0.7
LV mass index	1 g/m^2^	1.01 (1.00–1.01)	0.04
Log_10_ Angpt-2	1 ng/mL	4.53 (1.24–16.47)	0.02
hs- CRP	1 mg/L	1.00 (0.97–1.03)	0.9
Serum albumin	1 g/L	0.97 (0.90–1.05)	0.4
Background VC	–	0.52 (0.24–1.13)	0.1
Background AVD	–	3.11 (1.60–6.05)	0.001

*AVD* atherosclerotic vascular disease, *VC* valvular calcification, *hs-CRP* high sensitive C-reactive protein, *LDL* lower-density lipoprotein, *LV* left ventricular, *Angpt-2* angiopoietin-2

## Discussion

To our knowledge, this is the first prospective study investigating the correlation of circulating angiopoietin-2 with the MIAC syndrome, as well as the impact of Angpt-2 in predicting clinical outcomes of patients undergoing PD. We find that: (i) serum Angpt-2 levels closely link with MIAC syndrome in PD patients. (ii) high Angpt-2 levels independently predict fatal and non-fatal cardiovascular events in PD patients.

Although Angpt-1 is constitutively expressed throughout adult vessels providing a stabilization signal, Angpt-2 expression is observed only at sites of active vascular remodeling and neoangiogenesis [31]. We found that the serum Angpt-2 levels were comparable among Chinese PD patients with the study by David et al. [19] in PD patients, which was significantly higher than controls. It has been identified that endothelial Weibel–Palade bodies (WPBs) is the primary source of Angpt-2, which is dramatically up-regulated on endothelial cell activation, frequently observed in patients with CKD [32, 33]. In addition, it has been reported that nitric oxide has decreased availability in patients with CKD, which is known as important inhibitor of WPB exocytosis [34]. We hypothesize that the increased serum Angpt-2 levels we found in our patients might be caused by excess WPB exocytosis as a consequence of decrease nitric oxide production. Furthermore, in vitro, increased Angpt-2 expression has been observed on stimulation with high glucose and tumor necrosis factor-alpha (TNF-α) levels, both frequently observed in dialysis patients [16, 20]. Further study was needed to explore the underlying mechanism.

In present study, patients in the upper tertile of serum Angpt-2 not only had the highest CRP but also were the most malnourished. This suggests that Angpt-2 not only is a potent factor involving in angiogenesis as shown previously [13–15], but may also be useful in indentifying PD patients complicated with MIAC syndrome. It has been reported that circulating Angpt-2 is significantly correlated with CRP level in CKD patients and elevated Angpt-2 levels are strong predictors of long-term mortality in these patients [35]. In a dose-dependent manner, Angpt-2 competitively inhibits binding of Angpt-1 to Tie-2, followed by loss of vessel integrity, vascular leakage and induction of inflammatory gene expression [17]. The pro-inflammatory property of Angpt-2 was evidenced further by increasing expression of Angpt-2 by the TNF-α treatment and knockdown of Angpt-2 ameliorating TNF-α-induced apoptosis [36]. Even though high Angpt-2 and high CRP may represent the same biological event, our data suggest that Angpt-2 may be useful in further stratifying the severity of the MIAC syndrome in PD patients. In present study, serum Angpt-2 and albumin were not correlated, but patients in the upper tertile of Angpt-2 levels had the lowest level of nPCR, another important index of nutrition statue in PD patients (*p *< 0.001). It has been found that higher plasma Angpt-2 was closely related with severe malnutrition among patients with advances human immunodeficiency virus (HIV) infection [37]. The causal relationship between malnutrition and circulating Angpt-2 levels need further study.

Emerging data support the notion that circulating angiopoietins levels closely related with atherosclerotic diseases. Previous studies had found that circulating Angpt-2 levels were related with atherosclerosis as well as the degree of myocardial damage [38, 39]. In addition, high Angpt-2 and low Angpt-1 levels were positively associated with abnormal cardiac structure in stage 3–5 CKD patients [40] and circulating Angpt-2 was inversely related to glomerular filtration rate (GFR) and increased with advanced CKD [18]. It has been reported that Angpt-2 level is an indicator of vascular bed-specific atherosclerotic burden in dialysis patients, having correlation with degree of both coronary heart disease (CHD) and peripheral arterial disease (PAD) [19]. Furthermore, a study in children on chronic dialysis has found that serum Angpt-2 positively correlated with dialysis duration, as well as with an anti-angiogenic (high soluble vascular endothelial growth factor receptor-1 and low vascular endothelial growth factor-A) and pro-inflammatory (high urate, E-selectin, P-selectin and vascular cell adhesion molecule-1) milieu [21]. This is well in line with our findings that patients having all components of the MIAC syndrome had the highest serum Angpt-2. Those findings gave additional evidence that serum Angpt-2 might be an important biomarker of atherosclerotic diseases as well as vascular calcification, which might dependent on its role in inducing micro-inflammation.

In present study, we found that serum Angpt-2 was an independently predictive factor for fatal and non-fatal cardiovascular events when adjusting for components of MIAC syndrome and other parameters. A community-based study including 3220 participants had showed that increasing serum Angpt-2 concentrations were associated with higher risk for all-cause and cardiovascular mortality [41]. A recent report also linked outcome in CKD stages 3–5 patients to circulating serum Angpt-2 and found that baseline Angpt-2 levels was an independent predictor for major adverse cardiovascular events (MACE) as well as all-cause mortality [42]. As shown in our study, serum Angpt-2, being a biomarker of calcification and micro-inflammation, was indeed closely related to the MIAC syndrome. Previous study has found that MIAC syndrome strongly predicted all-cause mortality and cardiovascular deaths in PD patients [43]. Besides, a study among patients with type I diabetes had also found that Angpt-2 was a significant independent factor for atherosclerosis risk due to its role in vascular dysfunction and may enable the recognition of preclinical cardiac impairment [44]. Furthermore, circulating Angpt-2 was also a biomarker for heart failure in adults with congenital heart disease (ACHD), which was comparable to NT-proBNP [45]. The levels of Angpt-2 and sTie-2 were associated with the metabolic syndrome (MetS) [46]. Increasing circulating Angpt-2 levels was an independent predictor for fatal or non-fatal cardiovascular events in PD patients partly due to its close relationship with MIAC syndrome, and partly due to its role in endothelial activation. As endothelial cells are involved in many aspects of vascular biology, including barrier function, immune surveillance, blood clotting, and atherosclerosis, ongoing endothelial activation as manifested by persistent elevations in plasma Angpt-2 may predispose to a wide array of adverse outcomes. Whether it has causal relationship with mortality, especially the cardiovascular event in PD patients, requires further investigation.

Our study had several limitations that require consideration. Firstly, a single time point measurement of serum Angpt-2 was performed and may not reflect changes over time or time-averaged exposure. Secondly, the cross-sectional relationship observed between serum Angpt-2 and the components of MIAC syndrome, however strong, did not allow causal inferences and the relationship could be incidental. Thirdly, the inclusion of prevalent but not incident PD patients may introduce survival bias.

## Conclusions

In summary, our study shows a close association between serum Angpt-2 and cardiac valvular calcification, atherosclerosis, inflammation and malnutrition in PD patients. High circulating Angpt-2 levels is an independently predictive factor for fatal and non-fatal cardiovascular events in PD patients. Further study is needed to explore a possible mechanistic link between Angpt-2 and MIAC syndrome as well as cardiovascular events in patients undergoing PD treatment.

## Additional file

**Additional file 1: Figure S1.** (A) Serum Angpt-2 concentration in relation to presence or absence of valvular calcification (VC) and atheroscleroticvascular disease (AVD). (B) Serum Angpt-2 concentration in relation to the presence (I+ ve: CRP ≥ 5 mg/L) or absence (I− ve: CRP < 5 mg/L) of inflammation and presence (M+ ve: albumin < 30 g/L) or absence (M+ ve: albumin  ≥ 30 g/L) of malnutrition.

## Abbreviations

Angpt-2: angiopoietin-2
MIAC: malnutrition, inflammation, atherosclerosis and cardiac valvular calcification
PD: peritoneal dialysis
hs-CRP: high sensitive C-reactive protein
nPCR: normalized protein catabolic rate
BNP: brain natriuretic peptide
LDL: lower-density lipoprotein
LV: left ventricular
ESRD: end-stage renal disease
CVD: cardiovascular diseases
CRP: C-reactive protein
IL-6: interleukin-6
SGA: subjective global assessment
MIA: malnutrition, inflammation and atherosclerosis
VC: valvular calcification
Angpt-1: angiopoietin-1
CKD: chronic kidney diseases
DM: diabetes mellitus
AVD: atherosclerotic vascular disease
sTie-2: soluble Tie-2
ELISA: enzyme linked immunosorbent assay
iPTH: intact parathyroid hormone
HDL: high-density lipoprotein
ANOVA: one-way analysis of variance
WPBs: Weibel–Palade bodies
TNF-α: tumor necrosis factor-alpha
HIV: human immunodeficiency virus
GFR: glomerular filtration rate
CHD: coronary heart disease
PAD: peripheral arterial disease
MACE: major adverse cardiovascular events
ACHD: adults with congenital heart disease
NT-proBNP: N-terminal pro-B-type natriuretic peptide
MetS: metabolic syndrome
Ca × P: calcium × phosphorus product
