# Supplementary material for: Association of serum angiopoietin-2 with malnutrition, inflammation, atherosclerosis and valvular calcification syndrome and outcome in peritoneal dialysis patients: a prospective cohort study
Source: J Transl Med. 2018 Nov 16;16:312. doi: 10.1186/s12967-018-1687-0 (PMC6240212; doi:10.1186/s12967-018-1687-0)
Supplement: Supplementary file 1 — Additional file 1: Figure S1. (A) Serum Angpt-2 concentration in relation to presence or absence of valvular calcification (VC) and atheroscleroticvascular disease (AVD). (B) Serum Angpt-2 concentration in relation to the presence (I+ ve: CRP ≥ 5 mg/L) or absence (I− ve: CRP < 5 mg/L) of inflammation and presence (M+ ve: albumin < 30 g/L) or absence (M+ ve: albumin ≥ 30 g/L) of malnutrition. [file 12967_2018_1687_MOESM1_ESM.docx]

A

B

**Figure S1**. (A) Serum Angpt-2 concentration in relation to presence or absence of valvular calcification (VC) and atheroscleroticvascular disease (AVD). (B) Serum Angpt-2 concentration in relation to the presence (I+ ve: CRP ≥ 5 mg/L) or absence (I- ve: CRP < 5 mg/L) of inflammation and presence (M + ve: albumin< 30 g/L) or absence (M + ve: albumin ≥ 30 g/L) of malnutrition.
